# NRT1.1 Dual-Affinity Nitrate Transport/Signalling and its Roles in Plant Abiotic Stress Resistance

**DOI:** 10.3389/fpls.2021.715694

**Published:** 2021-08-23

**Authors:** Xian Zhi Fang, Shu Qin Fang, Zheng Qian Ye, Dan Liu, Ke Li Zhao, Chong Wei Jin

**Affiliations:** ^1^Key Laboratory of Soil Contamination Bioremediation of Zhejiang Province, State Key Laboratory of Subtropical Silviculture, Zhejiang A&F University, Zhejiang, China; ^2^State Key Laboratory of Plant Physiology and Biochemistry, College of Natural Resources and Environmental Science, Zhejiang University, Hangzhou, China

**Keywords:** abiotic stress, nitrate transporter 1.1, dual-affinity, nitrate transport, nitrate signalling

## Abstract

NRT1.1 is the first nitrate transport protein cloned in plants and has both high- and low-affinity functions. It imports and senses nitrate, which is modulated by the phosphorylation on Thr101 (T101). Structural studies have revealed that the phosphorylation of T101 either induces dimer decoupling or increases structural flexibility within the membrane, thereby switching the NRT1.1 protein from a low- to high-affinity state. Further studies on the adaptive regulation of NRT1.1 in fluctuating nitrate conditions have shown that, at low nitrate concentrations, nitrate binding only at the high-affinity monomer initiates NRT1.1 dimer decoupling and priming of the T101 site for phosphorylation activated by CIPK23, which functions as a high-affinity nitrate transceptor. However, nitrate binding in both monomers retains the unmodified NRT1.1, maintaining the low-affinity mode. This NRT1.1-mediated nitrate signalling and transport may provide a key to improving the efficiency of plant nitrogen use. However, recent studies have revealed that NRT1.1 is extensively involved in plant tolerance of several adverse environmental conditions. In this context, we summarise the recent progress in the molecular mechanisms of NRT1.1 dual-affinity nitrate transport/signalling and focus on its expected and unexpected roles in plant abiotic stress resistance and their regulation processes.

## Introduction

Nitrogen (N) is a primary constituent of proteins and nucleotides that are essential for life. Nitrogen accounts for approximately 2–5% of the total dry biomass of plants (Xu et al., 2012). Nitrate (NO_3_^−^) is a major source of nitrogen in most plants grown in agricultural and natural systems (Wang et al., 2018). As plants have adapted to variable soil nitrate concentrations, sophisticated nitrate transporter systems have evolved. During the past two decades, four families of nitrate transport proteins, namely, nitrate transporter 1 (NRT1), nitrate transporter 2 (NRT2), chloride channel (CLC), and slow anion channel associated homologues (SLAC/SLAH), have been identified in higher plants (Krapp et al., 2014). Among these, NRT1.1, which has multiple functions, is one of the most well-studied. Initially, NRT1.1 was characterised as a dual-affinity nitrate transporter involved in nitrate uptake by roots, as well as root-to-shoot nitrate translocation in *Arabidopsis* (Liu et al., 1999; Léran et al., 2013). Independent of its transport function, NRT1.1 was later shown to serve as a main nitrate sensor that regulates many aspects of physiological and developmental responses to nitrate, including regulating the expression levels of nitrate-related genes, modulating root system architecture, and relieving seed dormancy (Bouguyon et al., 2015). Moreover, NRT1.1 displays auxin transport activity, which relies largely on external nitrate availability in *Arabidopsis* (Maghiaoui et al., 2020a). In recent years, specific topics associated with the transport and sensing functions of NRT1.1 have been discussed in several excellent reviews (Sun and Zheng, 2015; Maghiaoui et al., 2020b; Vidal et al., 2020; Wang et al., 2020b). A series of studies on NRT1.1 have also provided new insights into its function in multiple abiotic stresses in plants. In this review, we briefly summarise the important milestones in the discovery process, dual-affinity features, and structural basis of the dual transport/sensing function of NRT1.1 in *Arabidopsis*. More importantly, we highlight the most recently characterised functions of NRT1.1 in plant abiotic stress resistance and the correlation between NRT1.1-mediated nitrate transport/signalling and different abiotic stresses, mainly in *Arabidopsis* (Table 1).

**Table 1:** Summary of the regulatory mechanism of NRT1.1 in abiotic stress resistance.

Abiotic stress types	NRT1.1 Function	The relation with NO_3_^−^ transport or signalling	Reference
H^+^	H^+^ toxicity induced NRT1.1-mediated H^+^-coupled NO_3_^−^ uptake, which in turn alleviated plant H^+^ stress by enhancing rhizosphere pH	NO_3_^−^ uptake	Fang et al. (2016)
Na^+^	NRT1.1 intensified Na^+^ accumulation in plants grown with NO_3_^−^ but entrapped plants in a Cl^−^-excess status under NH_4_^+^ conditions	NO_3_^−^ transport	Álvarez-Aragón and Rodríguez-Navarro, (2017); Liu et al. (2020)
Drought	Disruption of NRT1.1 in plants reduced nitrate accumulation in guard cells and did not cause nitrate-induced membrane depolarisation, leading to smaller stomatal opening	NO_3_^−^ transport	Guo et al. (2003)
Cd^2+^	Loss of NRT1.1 in plants led to decreased levels of Cd in NO_3_^−^-containing medium; NRT1.1-mediated NO_3_^−^ allocation to roots by coordinating Cd^2+^ accumulation in root vacuoles, facilitating Cd^2+^ detoxification of the wild type	NO_3_^−^ transport	Mao et al. (2014); Jian et al. (2018)
Zn^2+^	A lack of NRT1.1 function in plants led to the reduced accumulation of Zn in *nrt1.1* mutants under Zn stress, thereby enhancing Zn tolerance	NO_3_^−^ uptake	Pan et al. (2020)
Pb^2+^	The reduced Pb uptake in wild type was caused by the reduction of Pb bioavailability in the rhizosphere due to H^+^ consumption during NO_3_^−^ uptake of NRT1.1	NO_3_^−^ uptake	Zhu et al. (2019)
Low-K^+^	NRT1.1 participated in coordinating nitrate and potassium uptake and allocating plants under low-K^+^, which rely on the interactions between NRT1.1 and K^+^ channels/transporters located in the root epidermis-cortex and central vasculature	NO_3_^−^ transport	Fang et al. (2020)
NH_4_^+^	NH_4_^+^ toxicity was related to a nitrate-independent signalling function of NRT1.1 in *Arabidopsis*, characterised by reduced NH_4_^+^ accumulation and improved NH_4_^+^ metabolism, which may affect ethylene synthesis of *nrt1.1* mutants	NO_3_^−^ signalling	Hachiya and Noguchi, (2011); Jian et al. (2018)
P starvation	PHO2 functioned as an integrator of the N availability into the PSR because the effect of N on PSR is significantly affected in PHO2 mutants. PHO2 and NRT1.1 influence the transcript levels of each other	NO_3_^−^ signalling	Medici et al. (2019)
Fe deficiency	A lack of *NRT1.1* enhanced plant tolerance to Fe deficiency; the reduced accumulation of internal nitrate in *nrt1.1* mutants may impair the *FIT*-dependent Fe deficiency signalling pathway	NO_3_^−^ signalling	Liu et al. (2015)

### Discovery of NRT1.1

The active uptake of nitrate through membrane transporters *via* the roots is the first critical step in nitrogen acquisition. To date, many genes encoding nitrate transporters have been identified in higher plants. The first plant mutant defective in nitrate uptake, *chl1-1*, identified as early as 1978, showed impaired absorption of chlorate, a nitrate analogue that is toxic to plants (Doddema et al., 1978; Wen et al., 2017). However, these studies failed to isolate *CHL1*. In 1993, Tsay et al. successfully screened a new chlorate-resistant mutant that was an allele of *chl1-1* among a pool of T-DNA-tagged transgenic plants. Further analysis of the genomic DNA flanking the T-DNA insert revealed that the target gene was mapped to the top of chromosome 1, where *chl1-1* is located. Missing fragments of the *CHL1* mutant were then isolated from wild-type *Arabidopsis*. Thus, the *CHL1* gene was successfully cloned for the first time and had no significant identity to any other reported protein sequence until 1993. By comparing the predicted membrane topology with many other cotransporters in plants and animals, Tsay et al. (1993) proposed that *CHL1* may encode a nitrate transporter. To further determine the function of the *CHL1* protein, the authors engineered a *CHL1*-injected oocyte expression system which had a clear inward current of nitrate across the plasma membrane, especially at relatively low pH conditions (Tsay et al., 1993; Crawford and Glass, 1998). Therefore, this finding marks the first successful identification of the nitrate transport gene *NRT1.1* (*CHL1*) in plants.

### Dual-Affinity Function of NRT1.1

In response to fluctuations in external nitrate concentrations, two nitrate uptake systems have evolved in plants: a low-affinity transport system (LATS) and a high-affinity transport system (HATS), which are controlled through the NRT1 and NRT2 gene families, respectively (Wang et al., 2012). Interestingly, NRT1.1 is an exception, having both high- and low affinity for nitrate (Liu and Tsay, 2003). Switching between high- and low affinity of NRT1.1 is mediated *via* phosphorylation modification on a key threonine residue, Thr101 (T101). Recent structural analysis revealed that the phosphorylation of T101 not only induces dimer decoupling, but also increases structural flexibility within the membrane, thereby switching the NRT1.1 protein from a low- to high-affinity state (Tsay, 2014). Further structural and biochemical modelling has uncovered a bistable control of NRT1.1-mediated nitrate signalling by activating its upstream CBL9-CIPK23 complex in response to a wide range of fluctuating soil nitrate conditions (Rashid et al., 2019).

#### Contribution of NRT1.1 Dual-Affinity to Nitrate Uptake in Plants

NRT1.1 was first characterised as a low-affinity nitrate transporter (LAT), as disruption of NRT1.1 function in *nrt1.1* mutants led to a >80% decrease in nitrate uptake in sufficient nitrate (25mm KNO_3_) growth medium compared with that of the wild-type plants (Huang et al., 1996). Consistent with this result, a recent study by Ye et al. (2019) reported that the *nrt1.1* mutants showed approximately 50% less nitrate uptake than the wild type under 4mm nitrate conditions, indicating that the contribution of LATS of NRT1.1 at high nitrate supply was at least 50%. However, when nitrate levels were below 0.25mm, NRT1.1 was shown to act as a high-affinity nitrate transporter (HAT) and NRT1.1 was demonstrated to be responsible for approximately 75% of HATS in *Arabidopsis* (Liu et al., 1999). Subsequent analysis of nitrate uptake activity in plants and *Xenopus oocytes* revealed that NRT1.1 has a biphasic nitrate uptake kinetic feature, in which the affinity switch is regulated by the phosphorylation on the T101 residue of the NRT1.1 protein (Liu and Tsay, 2003; Rashid et al., 2018), and these findings provided the underlying operating mechanism of NRT1.1 dual-affinity activity. Notably, investigators in some later studies questioned the contribution of the HATS of NRT1.1 to nitrate uptake under low nitrate conditions (Glass and Kotur, 2013; Noguero et al., 2018); for example, functional disruption of NRT2.1 in plants resulted in a 96% reduction in the HATS influx of nitrate (Yong et al., 2010; Kotur et al., 2012), indicating that the contribution of the HATS of NRT1.1 at low nitrate supply was <4% of the wild-type uptake. Intriguingly, Ye et al. (2019) recently re-evaluated the role of NRT1.1 in nitrate uptake in *Arabidopsis* under low nitrate supply by generating a *nrt1.1*/*2.1*/*2.2* triple mutant that could eliminate the contributions of NRT2.1 and NRT2.2 on the HATS influx of nitrate. The *nrt1.1*/*2.1*/*2.2* triple mutant was found to have greater growth arrest and a lower rate of nitrate uptake than the *nrt2.1*/*2.2* double mutants in 0.2mm nitrate growth medium, suggesting that NRT1.1-mediated HATS is necessary for plant growth under low nitrate growth conditions. By subtracting the root nitrate uptake rate of the *nrt1.1*/*2.1*/*2.2* mutants from those of the *nrt2.1*/*2.2* mutants, the authors proposed that ~12% of the high-affinity nitrate uptake in plants was attributed to NRT1.1 in 0.2mm nitrate growth medium (Ye et al., 2019). Therefore, NRT1.1 is indispensable for maintaining plant growth under both high- and low nitrate growth conditions.

#### Structural Basis of NRT1.1 Dual-Affinity

With the aim of further illustrating how T101 phosphorylation switches the transport affinity of NRT1.1, researchers in two independent studies revealed the crystal structure of *Arabidopsis* NRT1.1, suggesting a potential structural significance for phosphorylation (Parker and Newstead, 2014; Sun et al., 2014). The NRT1.1 protein crystallises with two monomers (A and B) in each asymmetric unit which are almost identical to each other and adopt the canonical major facilitator superfamily fold. Each monomer is comprised of 12 transmembrane spanning alpha helices (TMHs) that form a clearly defined cavity that opens towards the cytoplasmic side (Sun and Zheng, 2015; Rashid et al., 2019), within which the substrate can bind. Therefore, unmodified NRT1.1 has an inward-facing conformational state. In the crystal, the phosphorylation site, T101, is located at the N-terminal end of one TMH and is entirely buried in a hydrophobic pocket that is directly adjacent to the dimer interface. Based on data from several analyses, Sun et al. (2014) proposed that NRT1.1 adopts a dimer configuration and functions as a low-affinity transporter, whereas phosphorylated NRT1.1 undergoes dimer decoupling and shows a high-affinity state. How the dimeric switch regulates the Michaelis constant (Km) of NRT1.1 remains unknown.

**Figure 1:**
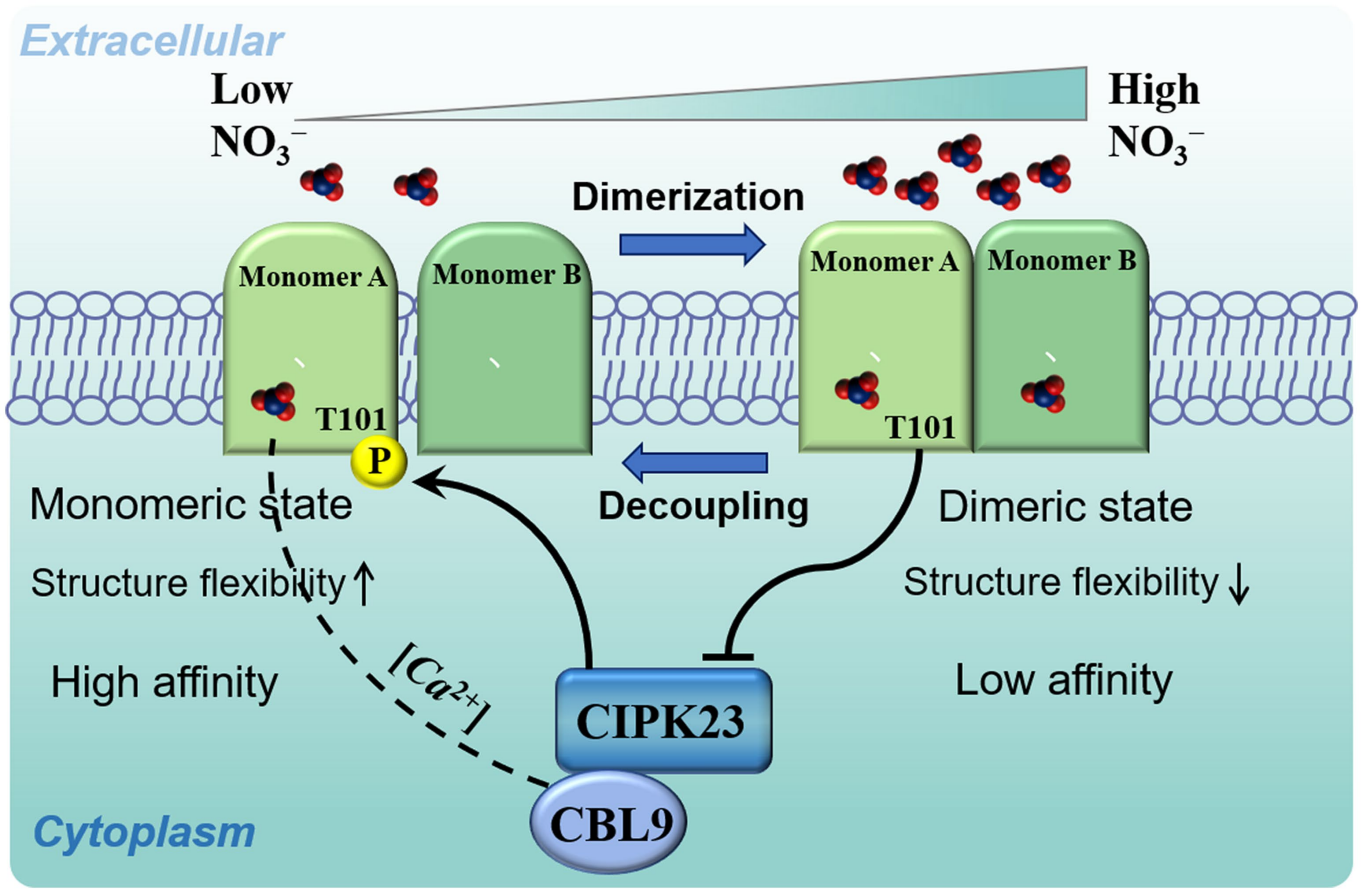
A model of NRT1.1-mediated biphasic control of nitrate signalling and transport. At low nitrate concentrations, nitrate binds only at the high-affinity site of monomer A, which induces asynchronous motions that initiate NRT1.1 dimer decoupling and priming of the Thr101 site for phosphorylation by the interactions with the CBL9-activated kinase, CIPK23. This phosphorylation eventually establishes a stable monomeric state of NRT1.1, which acts as a high-affinity nitrate transceptor. At high nitrate concentrations, nitrate binds to both monomers, which maintains synchronous motions that retain the dimeric state of NRT1.1 by attenuating the activity of the kinase, CIPK23, thereby regulating low-affinity nitrate signalling and transport.

In addition to decoupling the dimer, phosphorylation of T101 can alter the localised structural properties of the dimer (Parker and Newstead, 2014). To investigate the function of T101 phosphorylation, Parker and Newstead generated a Thr101Asp mutant, which can mimic permanent phosphorylation of NRT1.1. As predicted, the Thr101Asp mutant, as NRT1.1-101D, showed a lower melting temperature, indicating enhanced structural flexibility compared to the NRT1.1 protein of the wild type. Meanwhile, the nitrate transport rate of the Thr101Asp mutant was higher than that of the wild-type protein based on the liposome-based uptake assay. Thus, T101 phosphorylation increases the nitrate transport rate, which may result from the enhanced structural flexibility of the NRT1.1 protein. The seemingly contrasting conclusions of the two studies can, however, be reconciled—phosphorylation on T101 induces dimer decoupling, which might increase structural flexibility, thereby converting the low-affinity state of NRT1.1 to a high-affinity state (Figure 1; Tsay, 2014; Rashid et al., 2018).

#### Nitrate Binding in NRT1.1 and its Biphasic Adaptive Activity

A key question for the working mechanism of NRT1.1 is how can nitrate be recognised? The aforementioned studies on the NRT1.1 crystal implied that His356 is an important structural element for nitrate binding of NRT1.1, which was demonstrated by mutagenesis studies where H356A abolished nitrate uptake activity of NRT1.1 at high and low nitrate concentrations (Sun et al., 2014; Wen and Kaiser, 2018). Consistent with this finding, Rashid et al. (2018) carefully compared the nitrate-binding pocket composition of two monomers (A and B) in apo- and nitrate-bound crystal structures of NRT1.1, noting that nitrate binds to His356 and Thr360 through H-bonding in monomer A, and to His356 and Arg45 in monomer B. Compared with the apo-protein structure, in the nitrate-bounded protein structure, the T101 neighbourhood composition in monomer A differs by the residues Ala106 and Val163, and in protomer B, the composition differs by the residues Ala165. Furthermore, Ramachandran plot and electron density maps for NRT1.1 apo- and nitrate-bound protein showed that nitrate binding triggers large conformational changes of both the nitrate-binding residues and phosphorylation sites T101, enhancing asymmetries between the monomers, which bring a functional consequence that the affinity of monomer A has almost a 5-fold higher affinity than monomer B, indicating their differential roles in the nitrate binding of NRT1.1 (Pires and Ascher, 2016; Rashid et al., 2018). Further rigidity analysis of protein structure found that nitrate binding triggers more changes in chemical interactions in monomer A, resulting in the redistribution of rigid clusters of atoms, which form the largest rigid cluster (LRC) and interlink the nitrate-binding pocket and the phosphorylation site residues (Rashid et al., 2018, 2019). Such a rigid cluster has not been predicted in protomer B, indicating weak or absent allosteric communication between the binding and T101 sites. *In silico* mutational analyses in monomer A showed that the single amino acid mutant, Thr101Ala (which mimics the de-phosphorylated state of NRT1.1), breaks the rigid cluster that is responsible for allosteric communication into two distinct clusters, whereas the mutant Thr101Asp (which mimics the phosphorylated state of NRT1.1) maintains the intact allosteric rigid cluster. This finding is in parallel with the experimental result of Ho et al. (2009). Therefore, these results suggest that the priming of the T101 site in monomer A for the phosphorylation is allosterically triggered by the high-affinity nitrate binding, whereas in monomer B, such allosteric communication and priming are absent (Rashid et al., 2018, 2019).

The NRT1.1 protein functions as a toggle shift *via* the phosphorylation/dephosphorylation of T101, a functional switch for regulating nitrate signalling and transport. Nitrate binding to NRT1.1 is responsible for generating special calcium waves through the action of phospholipase C, and blocking the induction of these waves could severely influence several nitrate-induced responses (Riveras et al., 2015; Armijo and Gutiérrez, 2017). For this phosphorylation, activities of the CBL9-CIPK23 complex towards NRT1.1 appear to be dependent on these calcium waves (Ho et al., 2009; Léran et al., 2015). More recently, the dimerization switch of NRT1.1 was confirmed to play an important role in creating cytoplasmic calcium waves sensed by CBL9, which activates the kinase, CIPK23, at low nitrate concentrations, which is inhibited at high nitrate concentrations (Rashid et al., 2018, 2019). Because dimerization itself can change the binding affinity of NRT1.1, the relative intermonomer dynamics were demonstrated to have strong connections with dimer coupling/decoupling. At low external nitrate concentrations, nitrate binds only to the high-affinity monomer A, which induces significant changes in collective atomic motions and causes the loss of interface area and priming dimer decoupling. The resulting conformational dynamics also reorient the nitrate-channelling helices, inhibiting nitrate binding at low-affinity monomer B. Altogether, binding of nitrate at the high-affinity monomer initiates NRT1.1 dimer decoupling and priming of the T101 site for phosphorylation activated by CIPK23 at low nitrate concentrations. This monomeric state of NRT1.1 acts as a high-affinity nitrate transceptor. However, when nitrate binds to both monomers, the dimeric state of NRT1.1 is maintained, with concurrent attenuation of CIPK23 activity, thereby regulating low-affinity nitrate signalling and transport (Figure 1).

### Roles of NRT1.1 in Abiotic Stress and Their Relation to Nitrate Transport

**Figure 2:**
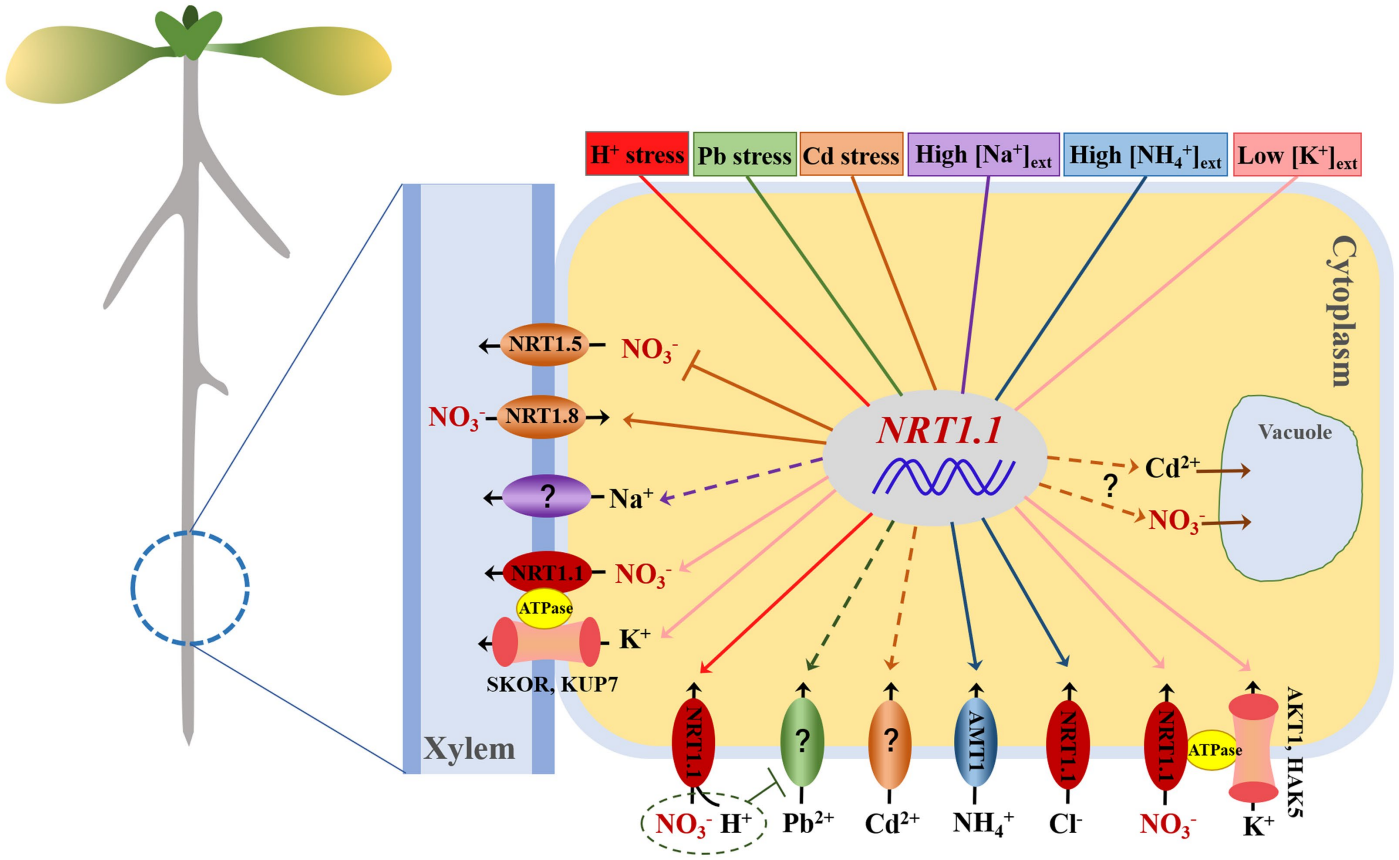
Schematic illustration of NRT1.1 nitrate transport in response to different stresses by mediating several transporters/channels. Proton toxicity (in red), lead stress (green), cadmium stress (orange), high external salt (purple), high external ammonium (blue), and low external potassium (pink). Arrows in solid lines and broken lines denote the demonstrated positive regulation and hypothetical regulation of transporters/channels by NRT1.1, respectively. Blunt arrows indicate negative regulation of targets by NRT1.1.

The uptake, accumulation, and assimilation of nitrate have long been observed to be closely associated with abiotic stress (Guo et al., 2003; Luo et al., 2012; Zhang et al., 2018). As the most studied nitrate transporter, NRT1.1 has been revealed to be responsible for most of the nitrate uptake of plants *via* roots and root-to-shoot nitrate translocation as well as nitrate transport in guard cells (Léran et al., 2013; Zhang et al., 2018). NRT1.1-mediated nitrate transport in different tissues mainly contributes to plant growth; however, it may also hint at an evolutionary adaptation of plants to environmental changes. In recent years, increasing evidence has suggested that NRT1.1 is extensively involved in resolving adverse environmental conditions (Table 1). NRT1.1 has been reported to use different mechanisms to regulate plant resistance to different stresses, some of which seem to have a potential connection (Figure 2). Here, we summarise the expected and unexpected roles of NRT1.1 in plant resistance to abiotic stresses and further discuss the relationship between these regulatory mechanisms and nitrate transport mediated by NRT1.1.

#### Proton Toxicity

NRT1.1 has been reported to contribute to the bulk of total nitrate uptake in roots *via* the mechanism of one nitrate ion and two protons symport across the plasmalemma (Huang et al., 1996; Wang et al., 2012). Recently, NRT1.1 was proposed to play an important role in plant tolerance to H^+^ toxicity. By examining the H^+^ tolerance of *nrt1.1* knockout mutants, an uptake- and sensing-decoupled mutant, *chl1-9* (which has reduced nitrate uptake but exhibits normal nitrate sensing activity; Ho et al., 2009), and wild-type plants, these *nrt1.1* mutants were found to have reduced H^+^ tolerance compared with the wild type, indicating that nitrate uptake activity was required for the NRT1.1-conferred H^+^ tolerance. Further experiments in these plants also revealed that NRT1.1-conferred H^+^ tolerance of plants is closely related to the enhanced rhizosphere pH as a consequence of the increased nitrate absorption stimulated by H^+^ toxicity (Fang et al., 2016; Feng et al., 2020). In conclusion, H^+^ in the rhizosphere induces H^+^-coupled NO_3_^−^ uptake by NRT1.1, thus altering the rhizosphere pH. Therefore, this function is largely attributable to the direct effect of NRT1.1 uptake activity. However, information on how plants perceive acid stress is still required in order to better understand the role of NRT1.1 in plant response to proton stress.

#### Drought and High Salt Stress

Drought and high salt are two major abiotic stresses that retard plant growth and reduce crop yield. Plants grown in nature have developed unique and overlapping resistance mechanisms in response to drought and salt stress (Zhu, 2016). Although NRT1.1 has been reported to participate in plant resistance to these two types of stress, their control mechanisms seem to have no intersection. Drought stress is well known to trigger the production of abscisic acid (ABA), which in turn leads to stomatal closure and induces the expression of several stress-related genes to acquire drought resistance in plants (Mittler and Blumwald, 2015; Jogawat et al., 2021). Nevertheless, NRT1.1-regulated plant resistance to drought might not be associated with ABA, as exogenous ABA application to leaves caused no significant difference in stomatal apertures between wild-type plants and *nrt1.1* mutants (Guo et al., 2003). NRT1.1 is also expressed in *Arabidopsis* guard cells. The *nrt1.1* mutants were found to have smaller stomatal apertures and thus more drought tolerance than wild-type plants grown in the medium with nitrate, which might be due to a lack of NRT1.1 and decreased nitrate accumulation in guard cells and failed to show nitrate-induced membrane depolarisation (Guo et al., 2003). This finding suggests that the inhibition of NRT1.1-mediated NO_3_^−^ transport into guard cells may enhance plant resistance to drought stress, but the mechanisms underlying this are still elusive. Notably, it was reported recently that ABA signalling negatively regulates nitrate acquisition *via* phosphorylation of NRT1.1 by SnRK2s in *Arabidopsis* under nitrogen deficiency (Su et al., 2021). Several researchers have also found that CIPK23 is involved in ABA responses (Léran et al., 2015; Reyes and Grégory, 2020; Su et al., 2021). Therefore, endogenous ABA might play an important role in modulating NRT1.1-mediated NO_3_^−^ transport during drought stress *via* two routes, including CIPK23 and SnRK2. Future work should concentrate on the molecular mechanisms connecting ABA to NRT1.1 under drought stress.

As the presence of nitrate enhances both root Na^+^ uptake and shoot Na^+^ accumulation in plants (Álvarez-Aragón et al., 2016), one or several nitrate transporters might modulate Na^+^ transport in plants. Although Na^+^ accumulation in the *nrt1.1* mutants was significantly lower than that in wild-type plants, this difference was abolished when nitrate was removed (Álvarez-Aragón and Rodríguez-Navarro, 2017). This finding indicates that NRT1.1 either partly mediates or modulates NO_3_^−^-dependent Na^+^ transport. However, a more recent study by Liu et al. (2020) proposed novel ideas of NRT1.1-conferred salt stress in plants. According to these researchers, several plant species fed NH_4_^+^ were more hypersensitive to NaCl stress and acquired more Cl^−^ and less Na^+^ than those fed NO_3_^−^. Further investigation of *Arabidopsis* showed that salt stress induced by the supply of NH_4_^+^ was abolished by the removal of Cl^−^ but was not mitigated by Na^+^ removal, implying that excess Cl^−^ rather than Na^+^ is responsible for NH_4_^+^-conferred salt hypersensitivity. Because NRT1.1 also participates in root Cl^−^ acquisition, NRT1.1 knockout in plants reduced their root Cl^−^ uptake and alleviated NH_4_^+^-aggravated salt stress in plants. Therefore, the potential mechanisms of NRT1.1-conferred salt stress in plants might be closely related to the form of nitrogen supplied to the growth medium. In brief, NRT1.1 intensifies Na^+^ accumulation in plants grown with NO_3_^−^ but entraps plants in a Cl^−^-excess status under NH_4_^+^ conditions. How NRT1.1 balances NO_3_^−^ and Cl^−^ uptake in response to salt stress under conditions of different NO_3_^−^ and NH_4_^+^ levels still needs to be explored.

#### Heavy Metals Stress

Heavy metals affect plant growth and development and lead to severe human health hazards through contaminated food chains. NRT1.1 has been reported to be involved in regulating plant resistance to several heavy metal stresses (Mao et al., 2014; Jian et al., 2018; Zhu et al., 2019; Pan et al., 2020). Mao et al. (2014) found that the loss of NRT1.1 in plants under Cd treatment increased biomass and caused less uptake of Cd in both roots and shoots in the presence of nitrate, whereas no difference was observed between the *nrt1.1* mutants and wild-type plants in the absence of nitrate. This finding indicates that the functional disruption of NRT1.1 reduces Cd uptake, which enhances Cd tolerance based on NO_3_^−^ uptake activity. However, Jian et al. (2018) reported that wild-type plants are more Cd tolerant than the *nrt1.1* mutants, as more Cd and nitrate are allocated to the vacuole of roots, which is correlated with transcript level repression of *NRT1.5* but upregulation of *NRT1.8*. The distinct expression levels of *NRT1.5* and *NRT1.8* in the wild-type and *nrt1.1* mutants also suggested that the expression of these two genes is regulated by *NRT1.1* (Gojon and Gaymard, 2010). This discrepancy may be related to the variance of nitrate or iron concentrations in growth conditions between the two experiments, which are believed to markedly affect Cd uptake by roots in many studies (Yang et al., 2016; He et al., 2017; Zhu et al., 2020). Although the two studies provide different, even partly conflicting, results regarding the role of NRT1.1 in mediating Cd stress response in *Arabidopsis*, both processes require the coordination of NO_3_^−^ transport.

Similarly, the indirect effect of NRT1.1 nitrate transport activity was found to play a role in plant resistance to Zn stress. The lack of NRT1.1 function in *nrt1.1* mutants led to reduced accumulation of Zn in both roots and shoots under Zn stress, suggesting that the modification of NRT1.1 activity might also enhance the Zn tolerance of plants in an NO_3_^−^ uptake-dependent manner (Pan et al., 2020). Notably, the mechanism by which NRT1.1 confers resistance to Pb stress in plants markedly differs from that of NRT1.1 in Cd and Zn stresses. Loss of NRT1.1 function in plants caused greater Pb toxicity and higher Pb accumulation in NO_3_^−^-sufficient growth medium. The reduced Pb uptake in wild-type plants was further found to result from the reduction of Pb bioavailability in the rhizosphere due to H^+^ consumption during NO_3_^−^ uptake by NRT1.1 (Zhu et al., 2019). In addition, exogenous application of low Mo in plants has been shown to induce the transcript levels of NRT1.1 (Liu et al., 2017). Collectively, these reports show that NRT1.1-associated strategies may be useful for manipulating the absorption and accumulation of heavy metals in plants; however, the chemical features of the heavy metals *per se* should be carefully considered. With respect to much of the progress concerning the molecular mechanisms of NRT1.1-regulated resistance to heavy metal stresses in *Arabidopsis*, the physiological relevance of these findings in crop species needs to be thoroughly studied.

#### Low-K^+^ Stress

Low potassium (K^+^) concentrations in most soils often limit plant growth (Maathuis, 2009). Although many potassium channels and transporters have been identified over the past few decades (Wang and Wu, 2017). the molecular mechanisms underlying potassium transport and regulation in plants require a more complete understanding. Recently, nitrate transporter 1.5 (NRT1.5), initially characterised as a pH-dependent bidirectional nitrate transporter, has been shown to be involved in K^+^ allocation in plants (Drechsler et al., 2015; Li et al., 2017). Fang et al. (2020) also found that the loss of NRT1.1 in *nrt1.1* mutants disturbs K^+^ uptake and root-to-shoot allocation, resulting in greater growth arrest under low K^+^ stress conditions. Further physiological and genetic evidence revealed that both the uptake and root-to-shoot allocation of K^+^ in wild-type plants require the expression of NRT1.1 in the root epidermis-cortex and central vasculature. NRT1.1-involved coordination of NO_3_^−^ and K^+^ uptake and allocation largely relied on the interactions between NRT1.1 and K^+^ channels/transporters located in the root epidermis-cortex and central vasculature. Given that the uptake rates of NO_3_^−^ and K^+^ are often found to be positively correlated (Coskun et al., 2016), the activity of nitrate transporters in roots may be affected by K^+^, as evidenced by the observation that appropriate K^+^ supply clearly increased the expression of NRT1.1 in roots (Xu et al., 2020). Notably, Fang et al. (2020) revealed that these K^+^ uptake-related interactions are dependent on an H^+^-consuming mechanism associated with the H^+^/NO_3_^−^ symport facilitated by NRT1.1. Nevertheless, NRT1.5-involved K^+^ loading into the xylem was verified to be only associated with its role as a proton-coupled H^+^/K^+^ antiporter (Li et al., 2017), which is not associated with NO_3_^−^ transport. However, the detailed molecular mechanisms of such interactions in root K^+^ uptake, xylem K^+^ loading with NO_3_^−^, and the involvement of NRT1.1 and K^+^ channels/transporters in this process are still unclear.

### Roles of NRT1.1 in Abiotic Stress and Their Relation to Nitrate Signalling

Despite the aforementioned abiotic stress, NRT1.1 also participates in a few other types of abiotic stress resistance, which may be related to nitrate signalling. However, the underlying mechanisms of the sensing function of NRT1.1, which confers resistance to abiotic stress, remain largely unclear.

#### Ammonium Toxicity

Ammonium (NH_4_^+^) can be utilised as a predominant nitrogen source in some plant ecosystems, but becomes toxic at high concentrations, especially when available as the sole nitrogen source (Gao et al., 2010; Ruan et al., 2016). The presence of an appropriate concentration of nitrate can clearly alleviate NH_4_^+^ toxicity in many plant species (Roosta and Schjoerring, 2007; Hachiya et al., 2011). However, NRT1.1-mediated nitrate uptake did not appear to play a positive role in plant tolerance to NH_4_^+^ toxicity, as the functional disruption of NRT1.1 in plants caused higher tolerance to high NH_4_^+^, and the application of nitrate did not enhance the ammonium tolerance of *nrt1.1* mutants (Hachiya and Noguchi, 2011). Therefore, a nitrate-independent function of NRT1.1 could exist. Jian et al. (2018) proposed that high NH_4_^+^ levels induced the activities of NADH-dependent glutamate dehydrogenase and glutamic-oxaloacetic transaminase in *NRT1.1* knockout mutants *chl1-1* and *chl1-5*, which reduced NH_4_^+^ accumulation and thus improved tolerance to NH_4_^+^ toxicity. Because the NRT1.1 P492L point mutant *chl1-9* retains normal function in nitrate signalling, the similar sensitivity symptoms of *chl1-9* and the wild type in response to high NH_4_^+^ indicate that the existence of the signalling function of NRT1.1 is sufficient to induce NH_4_^+^ toxicity. Given that the phosphorylation state and NRT1.1 protein levels in *chl1-9* are similar to those of the wild type, the decreased assimilation rate of NH_4_^+^ in wild-type plants could also occur in *chl1-9* mutants, which results in NH_4_^+^ toxicity (Hachiya and Noguchi, 2011; Jian et al., 2018). However, convincing experimental data are still needed. Another plausible interpretation of the different tolerance to NH_4_^+^ toxicity in *NRT1.1* knockout mutants *chl1-1* and *chl1-5* and NRT1.1 P492L point mutant *chl1-9* is that they may show different capacities for NH_4_^+^ uptake. The existence of NRT1.1 plays a positive role in inducing the expression of AMT1s and NH_4_^+^ uptake (Jian et al., 2018). Although whether this mechanism is indeed involved in *chl1-9* needs to be further confirmed by biological analyses, it is worth assuming that the NRT1.1 in *chl1-9* is likely involved in NH_4_^+^ uptake. As a common component, CIPK23 was previously shown to directly interact with and phosphorylate the ammonium transporters AMT1; 1/2 and nitrate transporter NRT1.1, modulating their activity (Ho et al., 2009; Straub et al., 2017; Tian et al., 2021). It has been shown that the CBL9-CIPK23 complex is inhibited by NRT1.1 dimer (Rashid et al., 2018), which implies that the altered phosphorylation state of NRT1.1 in *chl1-9* could affect the activity of AMT1 proteins under control of different nitrogen signals (Wu et al., 2019). Accordingly, the signalling function of NRT1.1 might play a positive role in mediating NH_4_^+^ uptake and accumulation. In addition, NRT1.1-related NH_4_^+^ toxicity has been shown to be associated with ethylene and auxin synthesis (Esteban et al., 2016; Jian et al., 2018). However, more studies are needed to elucidate how ethylene and auxin are involved in modulating the ammonium tolerance of *nrt1.1* mutants.

#### P and Fe Deficiency

Nutrient deficiency can seriously deter the normal growth of plants and consequently result in a reduction in crop yield (Shrestha et al., 2020). The mechanisms regulating plant responses to single nutrient stress have been documented over the past few decades (Wang and Wu, 2013; Tewari et al., 2021). However, much remains to be studied, especially if one specific component is selected as a molecular technique to improve the resistance of plants to different nutrient deficiency stresses. Interestingly, NRT1.1 has been shown to be involved not only in regulating the resistance of *Arabidopsis* to low-K^+^ stress, but also in responding to P and Fe nutrient deficiencies. In a study by Medici et al. (2015), an early nitrate-inducible transcription factor (TF), HRS1 and its close homologue HHO1, was reported to repress primary root growth caused by P deficiency, but only when nitrate is present, suggesting a complex regulation of N and P signals. In another recent study, Medici et al. (2019) found that the phosphate starvation response (PSR) can be actively controlled by N supply, and this process also relies on a combination of local and long-distance systemic nitrate signalling pathways. PHOSPHATE2 (*PHO2*) transcript accumulation is upregulated by nitrate depletion, which is dependent on NRT1.1. However, most PSR genes were not found to be regulated by nitrate in the *PHO2* mutants, indicating that *PHO2* integrates nitrate signals into the PSR. Furthermore, NRT1.1 was repressed by P starvation and PHO2 acted as a positive regulator of NRT1.1, as the transcript levels of *NRT1.1* in the *PHO2* mutant were lower than those in the wild type (Huang et al., 2013; Medici et al., 2015, 2019). These results provide important insights into the underlying molecular mechanism by which N and P signalling pathways interact.

Recently, several studies have demonstrated that the dependence of PSR on nitrate availability is conserved across a wide range of plant species (Hu et al., 2019; Medici et al., 2019; Wang et al., 2020a). In rice, high nitrate supply increased P acquisition and induced the transcript levels of P transporter (PT) genes and P starvation-induced (PSI) genes, which correlates with an increase biomass of rice. However, this nitrate induction of PSI genes was found to be abolished in mutants of the OsNRT1.1B transporter, the orthologue of AtNRT1.1 in rice, indicating that the nitrate-triggered P response is dependent on OsNRT1.1B function (Hu et al., 2019). Hu et al. further found that nitrate-stimulated interaction of OsNRT1.1B with OsSPX4 facilitates the ubiquitination and degradation of the P signalling repressor protein OsSPX4, which allows the release of OsPHR2 (Zhou et al., 2008), a master TF of phosphate signalling, into the nucleus and activates the transcription of P utilization genes. Importantly, OsSPX4 was also shown to interact with and control the activity of the master TF of nitrate signalling, OsNLP3, in rice. Therefore, nitrate-stimulated degradation of OsSPX4 activates expression of phosphate and nitrate uptake genes, ensuring a coordinated utilization of N and P in plants (Hu et al., 2019; Poza-Carrión and Paz-Ares, 2019). In addition, a nitrate-inducible, GARP-type transcription repressor 1.2 (NIGT1.2) was found to modulate P and nitrate uptake in response to P starvation in *Arabidopsis*. Under P deficiency conditions, NIGT1.2 directly upregulated the expression of the phosphate transporter genes *PHT1;1* and *PHT1;4* and downregulated transcription of *NRT1.1 via* binding to cis-elements in their promoters. The authors also identified a similar regulatory pathway in maize (Wang et al., 2020a). Collectively, these findings highlight the complexity of the nitrate and phosphate responses, with NRT1.1 having a crucial conserved role in modulating the interaction. Further studies are needed to investigate the relevant downstream signal transduction pathways of this N–P integrator.

Liu et al. (2015) reported that the lack of *NRT1.1* enhances plant tolerance to Fe deficiency stress; however, the expression of Fe acquisition related-genes *FRO2*, *IRT1,* and *FIT* was lower in the *nrt1.1* mutants than in wild-type plants under Fe-deficient conditions, indicating that the *FIT*-dependent Fe deficiency signalling pathway was not involved in *NRT1.1*-regulated Fe deficiency responses. Because nitrate functions as a nutrient and a signalling molecule (Krouk, 2017), it is conceivable that the reduced accumulation of internal nitrate in *nrt1.1* mutants may impair the *FIT*-dependent Fe deficiency signalling pathway. However, more detailed studies are needed to explore the mechanisms underlying the NRT1.1-regulated Fe deficiency responses. Overall, a clear link was found between NO_3_^−^ and P, K, or Fe in the transport and signalling cascade (of NO_3_^−^) coordinated *via* NRT1.1 in plants. However, an in-depth understanding of the effects of the crosstalk between nitrogen and one or more nutrients is still necessary, which is very important for understanding and engineering plant adaptive responses to a fluctuating nutritional environment.

## Perspective

The dual-affinity mode of nitrate transport is one of the most outstanding functions of NRT1.1. As a result, considerable efforts have been made to characterise the structural mechanisms regulating the switch between the two states of the NRT1.1 protein. Through structural and biochemical modelling, the dimerisation state and/or structural flexibility of NRT1.1 have been proposed to play a key role in the phosphorylation-governed affinity switch. Remarkably, the sensor function of NRT1.1 also exhibits a biphasic manner, which is regulated by the phosphorylation of T101, which is controlled by the kinase CIPK23. However, many important questions remain to be addressed to further understand this unique protein. For example, with the fluctuation of nitrate concentrations in the external environment, the maintenance of dynamic balance and transition between the signalling and transport functions, and whether NRT1.1 can synchronously activate the signalling and transport functions should be addressed in future studies. As nitrate only binds to high-affinity monomer A, which initiates NRT1.1 dimer decoupling and priming of the T101 site for phosphorylation by CIPK23 in a low nitrate concentration (Rashid et al., 2018), the signalling and transport functions of both monomers in NRT1.1 at different monomeric and dimerisation states should be systematically characterised. By disrupting the dimer interface (Robertson et al., 2010), a phosphorylation-independent NRT1.1 monomer mutant may be obtained. Further structural analyses of such mutants could help to determine whether monomer B in phosphorylated NRT1.1 is functional and how the intermonomer allostery affects the levels of cytosolic calcium waves. Another equally important question that requires precise clarification is whether the nitrate perception site is the same as the transport site.

Although NRT1.1 is believed to be preferentially responsible for nitrate transport and signalling, many extended roles that are involved in the regulation of diverse abiotic stresses have been determined. As previously mentioned, NRT1.1 plays a positive role in the resistance of *Arabidopsis* to H^+^, Pb^2+^, and low-K^+^ stress, and a negative role in modulating many types of stress, such as Cd^2+^, Zn^2+^, NH_4_^+^, high-Na^+^, and drought stress (Figure 2). The reason why NRT1.1 can play multiple physiological roles and whether it simultaneously mediates these stress processes needs to be elucidated. The cation-anion balance process seems to be the most common mechanism whereby NRT1.1-mediated NO_3_^−^ transport modulates the synergetic transport of cations (such as H^+^, K^+^, Cd^2+^, Zn^2+^, and Na^+^), which theoretically might depend on the cooperation between anion transporters/channels and cation transporters/channels (Figure 2). However, there is as yet no molecular evidence for direct protein–protein interactions in this regard. Remarkably, a common signalling module, the CBL9-CIPK23 complex, has previously been shown to modulate the transport activities of AKT1, TPK (K^+^ channel), HAK5 (K^+^ transporter), IRT1 (Fe^2+^/Cd^2+^/Zn^2+^ transporter), AMT1.1/2 (NH_4_^+^ transporter), and NRT1.1 (NO_3_^−^ transporter), as well as the activity of FRO2 (ferric-chelate reductase), in several studies (Ragel et al., 2015; Tian et al., 2016; Straub et al., 2017; Dubeaux et al., 2018; Tang et al., 2020). Regulation of nitrate and cation transporters/channels by the same kinase CIPK23 supports the aforementioned speculation that the interactions might be coordinated, or at least partially coordinated, at the molecular level. In addition, CIPK23 has also been shown to participate in the drought stress response and in the regulation of ABA responsiveness of guard cells during their closure and opening *via* phosphorylation and triggering the opening of the guard cell anion channels SLAC1/SLAH3 (Maierhofer et al., 2014; Reyes and Grégory, 2020). It has been reported that the CBL9-CIPK23 complex is inhibited by the dimer coupling state of NRT1.1 at high nitrate concentrations (Rashid et al., 2019), which means that it also influences the transport of other ions or the responses to certain stresses. However, much work is still needed, making use of biochemical and structural approaches to master the functional specificities that allow a single protein to regulate such diverse abiotic stresses.

NRT1.1 has been found to be expressed in the epidermis-cortex and central cylinder of mature roots as well as guard cells of shoots (Guo et al., 2003; Fang et al., 2020). Future studies should focus on the specific functions that have been ascribed to NRT1.1 in different tissues for the regulation of plant tolerance to certain environmental stresses. As NRT1.1 can act as a transceptor by sensing variations in extracellular nitrate concentrations to modulate its biphasic adaptive state (Rashid et al., 2019), it could also play a role in sensing nitrate concentrations in different organs. However, the signalling function of NRT1.1 in plant tissues in response to environmental changes remains unclear. As the overlapping resistance processes of NRT1.1 in response to different stresses were found in different studies, the future efforts are needed to systematically investigate its detailed mechanisms in regulating a combination of two or more different abiotic stresses, which may be expected to enhance plant resistance to naturally occurring environmental conditions.

To date, most advances in understanding the molecular mechanisms of NRT1.1, which regulates plant tolerance to abiotic stress, have been achieved in controlled unique laboratory conditions or a certain genotype of model plants. In rice and maize, homologues of NRT1.1 have been characterised and revealed to have nitrate transport activity, indicating a conserved function of NRT1.1 in nitrate transport across different plant species (Hu et al., 2015; Wen et al., 2017; Wang et al., 2020a). In future, the ideal NRT1.1-related traits identified in *Arabidopsis* will be expected to be transferred to crops and subsequently produced *via* myriad molecular biology methods.

## Author Contributions

XF wrote the first draft and edited the manuscript. CJ added content and edited the manuscript. All authors contributed to the article and agreed to the submitted version.

## Conflict of Interest

The authors declare that the research was conducted in the absence of any commercial or financial relationships that could be construed as a potential conflict of interest.

## Publisher’s Note

All claims expressed in this article are solely those of the authors and do not necessarily represent those of their affiliated organizations, or those of the publisher, the editors and the reviewers. Any product that may be evaluated in this article, or claim that may be made by its manufacturer, is not guaranteed or endorsed by the publisher.
